# Remdesivir and molnupiravir had comparable efficacy in lung transplant recipients with mild-to-moderate COVID-19: a single center experience

**DOI:** 10.3389/frtra.2024.1408289

**Published:** 2024-07-04

**Authors:** Deepika Razia, Devika Sindu, Lauren Cherrier, Katherine Grief, Rajat Walia, Sofya Tokman

**Affiliations:** ^1^ Norton Thoracic Institute, St. Joseph’s Hospital and Medical Center, Phoenix, AZ, United States; ^2^ Department of Pulmonary Disease and Transplantation, Creighton University Health Sciences Phoenix Campus, Phoenix, AZ, United States; ^3^ Department of Pharmacy, University of Kentucky, Lexington, KY, United States

**Keywords:** remdesivir, molnupiravir, lung transplantation, mild to moderate COVID-19, clinical outcomes, antiviral therapy, efficacy

## Abstract

**Introduction:**

Remdesivir (REM) and molnupiravir (MOL) are commonly used to treat lung transplant recipients (LTRs) with COVID-19; however, the clinical efficacy of these medications is yet to be compared. In this retrospective cohort study, we compared the clinical outcomes between LTRs with mild-to-moderate COVID-19 treated with REM and those treated with MOL.

**Methods and Results:**

Between March 2020 and August 2022, 195 LTRs developed COVID-19 at our center. After excluding 82 who presented with severe disease requiring hospitalization, the remaining 113 were included in the analysis: 54 did not receive antiviral treatment, 30 were treated with REM, and 29 were treated with MOL. Adjusted multivariable logistic regression analysis showed similar rates of hospitalization (adjusted odds ratio (aOR) 1.169, [95% confidence interval (95% CI) 0.105–12.997, *p* = 0.899], ICU admission (aOR 0.822, 95% CI 0.042–16.220, *p* = 0.898), mechanical ventilation (aOR 0.903, 95% CI 0.015–55.124, *p* = 0.961), and COVID-19-related mortality (aOR 0.822, 95% CI 0.042–16.220, *p* = 0.898) between LTRs treated with REM and those treated with MOL for mild-to-moderate COVID-19, irrespective of SARS-CoV-2 strain.

**Conclusion:**

MOL may be a suitable alternative to REM to treat LTRs with mild-to-moderate COVID-19, and the choice of antiviral therapy can be driven by practical considerations such as route of administration and drug availability.

## Introduction

Lung transplant recipients (LTRs) are at increased risk of developing severe COVID-19 due to a combination of advanced immunosuppression and concomitant comorbidities such as diabetes, kidney disease, and advanced age (1). They also have a reduced response to vaccination (2–6), rapidly waning immunity after primary infection, and a high rate of healthcare utilization that may augment exposure to SARS-CoV-2, the virus that causes COVID-19. Thus, effective antiviral therapy is paramount to reducing COVID-19-related morbidity and mortality in this vulnerable patient population.

Antiviral therapies directed against SARS-CoV-2 approved for use in the United States include remdesivir (REM), molnupiravir (MOL), and nirmatrelvir–ritonavir; however, nirmatrelvir–ritonavir is not commonly prescribed to LTRs due to significant drug-drug interactions. The U.S. Food and Drug Administration (FDA) provided Emergency Use Authorization for REM in May 2020 and MOL in December 2021; both drugs subsequently gained FDA approval and have been widely used to treat patients with COVID-19. REM is administered intravenously and thus requires either hospitalization or access to an outpatient infusion site with appropriate staffing and infection control measures. MOL, on the other hand, is an oral medication that can be readily self-administered on an outpatient basis. Although MOL is far more convenient for non-hospitalized patients, its efficacy may be inferior to REM (7), and its efficacy in LTRs with COVID-19 is unknown. Thus, we compared the morbidity and mortality between LTRs with mild-to-moderate COVID-19 treated with REM and those treated with MOL. We then conducted multivariable logistic regression analyses to adjust for additional risk factors driving hospitalization, intensive care unit (ICU) admission, mechanical ventilation, and COVID-19-related mortality among these patients, including SARS-CoV-2 strain, vaccination, and monoclonal antibody therapy.

## Patients and methods

This is a single-center, retrospective cohort study of LTRs diagnosed with COVID-19, approved by the Institutional Review Board at our institution with a waiver of patient consent (PHX-21-500-198-73-18 dated 01/08/2021; continuing review applications were submitted and subsequently approved on 10/12/2021, 02/08/2022, 09/11/2022, and 10/11/2022). All patient care was carried out under strict compliance with the International Society of Heart and Lung Transplantation ethics statement and the Declaration of Helsinki.

LTRs who developed mild-to-moderate COVID-19, defined as symptomatic COVID-19 without indication for hospitalization, between March 2020 and August 2022 at our center were included. Those with severe disease requiring hospitalization were excluded. Patients were not treated with REM or MOL if they were unable to access antivirals or if symptoms of COVID-19 developed more than a week prior to contacting our transplant center. We stratified the patients into three study groups based on their treatment: no antiviral therapy, REM, and MOL. The primary outcome was hospitalization for COVID-19 within 60 days of diagnosis, and the secondary outcomes were ICU admission, mechanical ventilation, and COVID-19-related mortality.

## Statistical analysis

Data were expressed as count (percentage) or median (interquartile range). The independent-samples Mann-Whitney U test was used to compare continuous variables, and Fischer's exact test or chi-squared analysis was used to compare categorical variables. The Kaplan-Meier with log-rank test was used for unadjusted comparisons of COVID-19-related hospitalization and post-COVID-19 1-year survival between the three study groups. Univariate logistic regression analyses were conducted to compare the impact of REM and MOL, SARS-CoV-2 strain, COVID vaccination, pre-exposure prophylaxis (PrEP) with tixagevimab and cilgavimab, monoclonal antibody therapy, and reduction/cessation of mycophenolate mofetil on the study outcomes. A multivariable logistic regression model was then developed by entering antiviral therapy and SARS-CoV-2 strain as fixed covariates and including variables with *p* < 0.2 on univariate analysis with backward stepwise elimination based on the likelihood ratio test to report adjusted odds ratios and 95% confidence intervals of the variables that remained in the final model. All tests were two-sided and a *p*-value < 0.05 was considered statistically significant. Statistical analyses were performed using IBM SPSS Statistics v.29 (IBM Corp., Armonk, NY).

## Results

Between March 2020 and August 2022, 195 LTRs developed COVID-19 at our center. After excluding 82 who presented with severe disease requiring hospitalization, the remaining 113 were included in the analysis: 54 were not treated with antivirals, 30 were treated with REM, and 29 were treated with MOL. All three study groups were comparable in terms of baseline clinical characteristics including age at COVID-19 diagnosis, gender, pre-COVID-19 body mass index, type of LT (single vs. double), and time from LT to COVID-19 diagnosis (Table 1). Despite these similarities, the group of LTRs without antiviral treatment was enriched with patients who had very mild COVID-19 as they did not test for COVID-19 or contact the transplant center in a timely manner. In addition, there were significant differences in the circulating SARS-CoV-2 strain, with the original strain dominating the REM group (53.3%), and the Omicron strain dominating the untreated (61.1%) and MOL groups (96.6%) (*p* = 0.002 and *p* < 0.001, accordingly).

**Table 1:** Baseline characteristics, COVID-19 therapies, and unadjusted clinical outcomes in lung transplant recipients with COVID-19 who were not treated with antiviral therapy and those treated with remdesivir or molnupiravir.

Variables	LTRs treated with remdesivir (REM) *N* = 30	LTRs treated with molnupiravir (MOL) *N* = 29	*P*-value REM vs. MOL	LTRs not treated with antivirals^a^ *N* = 54	*P*-value no antivirals vs. REM	*P*-value no antivirals vs. MOL
Baseline clinical characteristics						
Age at COVID-19 diagnosis, years^b^	65.7 (57.2, 69.9)	68.9 (59.1, 72.4)	0.219	62.6 (57.7, 71.4)	0.889	0.225
Sex, male	18 (60.0)	18 (62.1)	1.000	32 (59.3)	1.000	1.000
Pre-COVID-19 BMI, kg/m^2^^b^	27.8 (24.3, 32.4)	25.6 (23.1, 30.8)	0.259	25.8 (21.5, 29.9)	0.081	0.724
Bilateral LT	29 (96.7)	29 (100.0)	1.000	52 (96.3)	1.000	0.540
Time from LT to COVID-19 diagnosis, months^b^	44.3 (16.3, 65.7)	31.9 (16.0, 66.8)	0.671	38.4 (20.0, 66.5)	0.896	0.613
Dominant circulating SARS-CoV-2 Strain						
Original	16 (53.3)	0 (0.0)	**<0**.**001**	8 (14.8)	**<0**.**001**	**0**.**002**
Alpha	5 (16.7)	0 (0.0)		9 (16.7)		
Delta	5 (16.7)	1 (3.4)		4 (7.4)		
Omicron	4 (13.3)	28 (96.6)		33 (61.1)		
COVID-19 vaccination and pre-exposure prophylaxis (PrEP)						
≥2 doses of mRNA vaccines	7 (23.3)	29 (100.0)	**<0**.**001**	35 (64.8)	**<0**.**001**	**<0**.**001**
PrEP with tixagevimab and cilgavimab	0 (0.0)	14 (48.3)	**<0**.**001**	7 (13.0)	**0**.**047**	**0**.**001**
COVID-19 therapy						
Monoclonal antibodies	5 (16.7)	23 (79.3)	**<0**.**001**	31 (57.4)	**<0**.**001**	0.056
Reduced/stopped anti-proliferative agent	16 (53.3)	28 (96.6)	**<0**.**001**	47 (87.0)	**0**.**001**	0.251
Unadjusted clinical outcomes						
Hospitalization	14 (46.7)	5 (17.2)	**0**.**025**	13 (24.1)	**0**.**050**	0.582
ICU admission	9 (30.0)	2 (6.9)	**0**.**042**	2 (3.7)	**0**.**001**	0.609
Mechanical ventilation	4 (13.3)	1 (3.4)	0.353	1 (1.9)	0.053	1.000
COVID-19 mortality	9 (30.0)	2 (6.9)	**0**.**042**	1 (1.9)	**<0**.**001**	0.278

Data expressed as count (percentage) unless otherwise specified; ^a^REM or MOL; ^b^data expressed as median (interquartile range).

LTRs, lung transplant recipients; REM, remdesivir; MOL, molnupiravir; BMI, body mass index; COVID-19, coronavirus disease 2019; LT, lung transplant; ICU, intensive care unit. Bold indicates statistically significant values with a *p* ≤ 0.05.

Compared to REM-treated LTRs, LTRs without antiviral treatment were more likely to have had ≥2 doses of mRNA vaccines, PrEP with tixagevimab and cilgavimab, monoclonal antibody therapy, and a reduction of immunosuppression at the time of COVID-19 diagnosis. In contrast, compared to MOL-treated patients, untreated patients were less likely to be vaccinated and less likely to have received PrEP as this group included patients that contracted COVID before these preventative therapies became available. Untreated patients were also less likely to receive monoclonal antibodies, likely due to a combination of lack of access and being outside of the therapeutic window. In addition, LTRs in the MOL group were more likely than those in the REM group to be vaccinated with ≥2 doses of an mRNA vaccine (100% vs. 23.3%, *p* < 0.001) and to have received pre-exposure prophylaxis (PrEP) with tixagevimab and cilgavimab (48.3% vs. 0%, *p* < 0.001). Lastly, LTRs in the MOL group were more likely to be treated with monoclonal antibodies (79.3% vs. 16.7%, *p* < 0.001) and to undergo a reduction or cessation of anti-proliferative medications at COVID-19 diagnosis than those in the REM group (96.6% vs. 53.3%, *p* < 0.001; Table 1).

**Figure 1:**
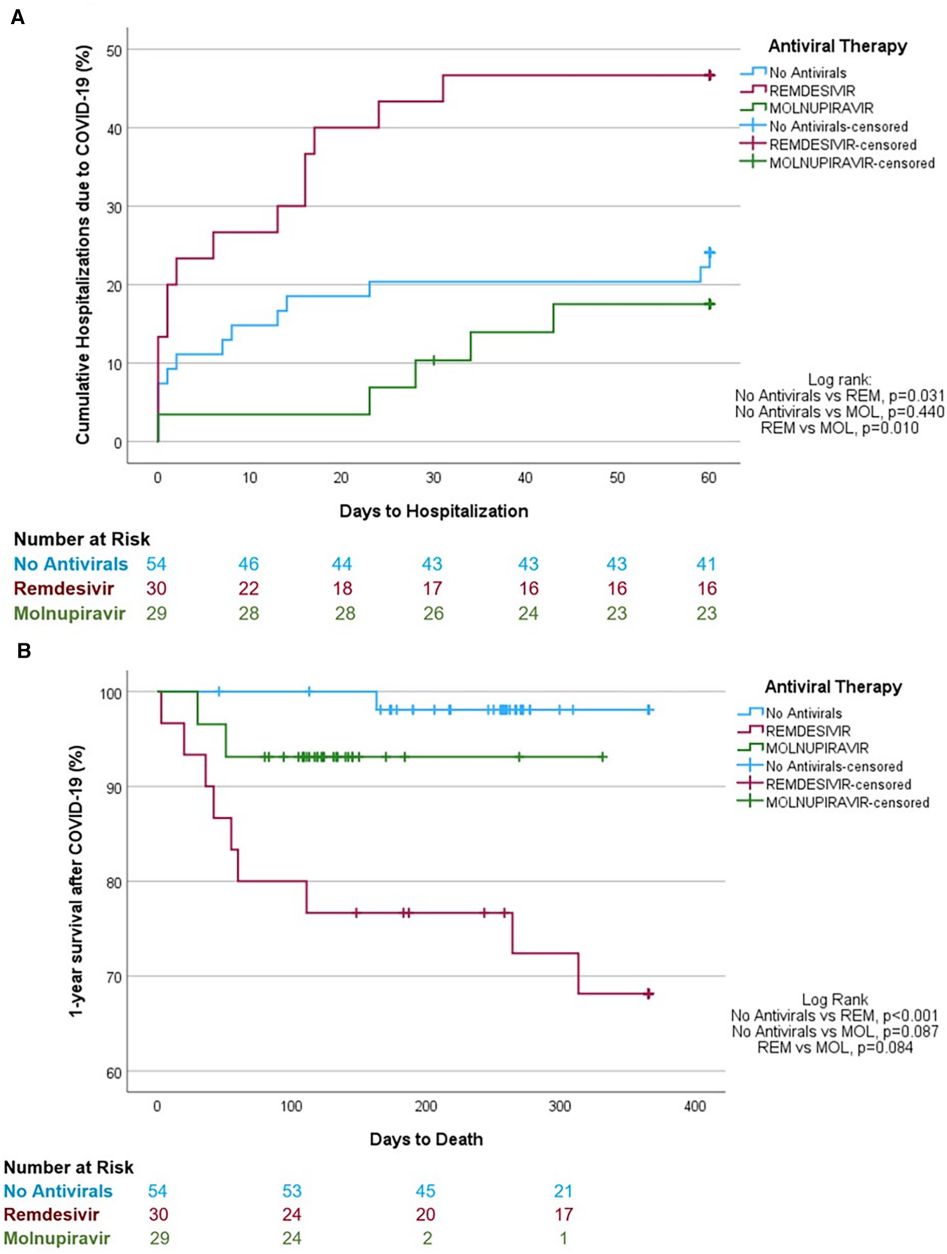
Unadjusted Kaplan-Meier analyses. (**A**) Hospitalization rates of lung transplant recipients with mild-to-moderative COVID-19 not treated with antiviral therapy or treated with remdesivir or molnupiravir. (**B**) 1-year survival estimates after COVID-19 diagnosis in lung transplant recipients not treated with antiviral therapy or treated with remdesivir or molnupiravir.

The unadjusted analysis shows that LTRs in the MOL group were significantly less likely to require hospitalization and ICU admission and were less likely to die of COVID-19 than LTRs in the REM group (Table 1). In addition, LTRs that were not treated with antivirals were less likely to require hospitalization and ICU admission and were less likely to die of COVID-19 than LTRs in the REM group, but had similar clinical outcomes to those treated with MOL. Lastly, LTRs that contracted the Omicron strain had similar clinical outcomes irrespective of whether they received no antiviral therapy or MOL (Table 2). Kaplan-Meier analysis revealed that LTRs treated with MOL had lower rates of hospitalization (log-rank *p* = 0.010; Figure 1A) and trended toward a higher likelihood of survival after COVID-19 (*p* = 0.084; Figure 1B) than those treat with REM. Similarly, LTRs that were not treated with antivirals had lower rates of hospitalization (log-rank *p* = 0.031; Figure 1A), a higher likelihood of survival than those treated with REM (log-rank *p* = 0.001; Figure 1B), and trended toward a higher likelihood of survival than those treated with MOL (log-rank *p* = 0.087; Figure 1B).

**Table 2:** Unadjusted clinical outcomes in lung transplant recipients with the Omicron strain who were not treated with antiviral therapy and those treated with molnupiravir.

Unadjusted clinical outcome	LTRs with Omicron treated with MOL *N* = 28	LTRs with Omicron not treated with antivirals *N* = 33	*P*-value
Hospitalization	5 (17.9)	5 (15.2)	1.000
ICU admission	2 (7.1)	2 (6.1)	1.000
Mechanical ventilation	1 (3.6)	1 (3.0)	1.000
COVID-19 mortality	2 (7.1)	1 (3.0)	0.589

Data expressed as count (percentage) unless otherwise specified; LTRs, lung transplant recipients; REM, remdesivir; MOL, molnupiravir; COVID-19, coronavirus disease 2019; ICU, intensive care unit.

A univariate logistic regression analysis was conducted for the variables with significant differences between the REM-treated and MOL-treated groups at baseline (Table 1) to evaluate the impact of these factors on COVID-19-related hospitalization, ICU admission, mechanical ventilation, and mortality (Table 3). On univariate analysis, the odds of hospitalization were significantly reduced by treatment with MOL compared to REM, contracting the Omicron strain rather than the earlier viral strains, vaccination with ≥2 doses of an mRNA vaccine, and PrEP (Table 3). The odds of ICU admission were significantly reduced by treatment with MOL compared to REM and contracting the Omicron strain rather than the earlier strains. Finally, the odds of COVID-19-related mortality were significantly reduced by treatment with MOL compared to REM, contracting the Omicron strain rather than earlier strains, and monoclonal antibody therapy. Vaccination likely reduced the odds of dying from COVID-19, however, the results were not statistically significant (*p* = 0.073; Table 3).

**Table 3:** Univariate and multivariable logistic regression analysis to evaluate hospitalization, ICU admission, mechanical ventilation, and COVID-19-related mortality in lung transplant recipients treated with remdesivir or molnupiravir.

Variable	Univariate analysis			Multivariable analysis		
	Odds ratio	95% confidence interval	*P*-value^a^	Adjusted odds ratio	95% confidence interval	*P*-value
Hospitalization						
Molnupiravir (reference: remdesivir)	0.238	0.072–0.791	**0**.**019**	1.169	0.105–12.997	0.899
SARS-CoV-2 strain, Omicron (reference: pre-Omicron)	0.172	0.051–0.581	**0**.**005**	0.151	0.014–1.631	0.119
≥2 doses of mRNA vaccines (reference: unvaccinated)	0.312	0.100–0.969	**0**.**044**	–	–	–
PrEP with tixagevimab and cilgavimab (reference: no PrEP)	0.115	0.014–0.961	**0**.**046**	–	–	–
Monoclonal antibody therapy (reference: no MAb therapy)	0.727	0.242–2.188	0.571	–	–	–
Reduced/stopped antiproliferative agent (reference: no change in antiproliferative agent	0.429	0.128–1.440	**0**.**171**	–	–	–
ICU admission						
Molnupiravir (reference: remdesivir)	0.173	0.034–0.886	**0**.**035**	0.822	0.042–16.220	0.898
SARS-CoV-2 strain, Omicron (reference: pre-Omicron)	0.133	0.026–0.687	**0**.**016**	0.157	0.008–2.985	0.218
≥2 doses of mRNA vaccines (reference: unvaccinated)	0.286	0.073–1.121	**0**.**073**	–	–	–
Monoclonal antibody therapy (reference: no MAb therapy)	0.345	0.082–1.460	**0**.**148**	–	–	–
Reduced/stopped antiproliferative agent (reference: no change in antiproliferative agent)	0.520	0.128–2.111	0.361	–	–	–
Mechanical ventilation						
Molnupiravir (reference: remdesivir)	0.232	0.024–2.214	0.204	0.903	0.015–55.124	0.961
SARS-CoV-2 strain, Omicron (reference: pre-Omicron)	0.185	0.019–1.772	**0**.**143**	0.202	0.003–12.064	0.443
≥2 doses of mRNA vaccines (reference: unvaccinated)	0.955	0.147–6.199	0.961	–	–	–
Monoclonal antibody therapy (reference: no MAb therapy)	0.250	0.026–2.385	0.228	–	–	–
Reduced/stopped antiproliferative agent (reference: no change in antiproliferative agent)	0.476	0.072–3.164	0.442	–	–	–
COVID-19-related mortality						
Molnupiravir (reference: remdesivir)	0.173	0.034–0.886	**0**.**035**	0.822	0.042–16.220	0.898
SARS-CoV-2 strain, Omicron (reference: pre-Omicron)	0.133	0.026–0.687	**0**.**016**	0.157	0.008–2.985	0.218
≥2 doses of mRNA vaccines (reference: unvaccinated)	0.286	0.073–1.121	**0**.**073**	–	–	–
Monoclonal antibody therapy (reference: no MAb therapy)	0.188	0.037–0.964	**0**.**045**	–	–	–
Reduced/stopped antiproliferative agent (reference: no change in antiproliferative agent)	0.520	0.128–2.111	0.361	–	–	–

^a^ Antiviral therapy and SARS-CoV-2 strain were entered as fixed covariates. Variables with *p* < 0.2 on univariate analysis were then included in the multivariable analysis with backward stepwise elimination to obtain the final model.

ICU, intensive care unit; PrEP, pre-exposure prophylaxis; MAb, monoclonal antibody; MMF, mycophenolate mofetil.

None of the LTRs who required ICU admission or mechanical ventilation and none of the LTRs who died of COVID-19 received PrEP with tixagevimab and cilgavimab, therefore, PrEP was excluded from these portions of the analysis. Bold values indicate *p* < 0.2 and therefore included in multivariate analysis.

A multivariable logistic regression model was then developed by entering REM or MOL therapy and SARS-CoV-2 strain as fixed covariates and including the variables with *p* < 0.2 on univariate analysis with backward stepwise elimination to obtain the final model. Strikingly, the odds of hospitalization, ICU admission, mechanical ventilation, and COVID-19-related mortality were not impacted by any of the therapies that were statistically significant on univariate analysis. On adjusted analysis, there were no differences in the need for hospitalization, ICU admission, mechanical ventilation, or mortality between LTRs with mild-to-moderate COVID-19 treated with REM and those treated with MOL (Table 3).

## Discussion

The COVID-19 pandemic has had wide-ranging implications within the field of lung transplantation and has had a tremendous effect on LTRs, LT programs, hospitals, and healthcare workers. The scientific community responded to this threat with the rapid development of mRNA vaccines, monoclonal antibodies, and antiviral therapies including REM and MOL, which are the foci of this analysis. Mutations within the spike protein of SARS-CoV-2 have led to changes in viral virulence, transmissibility, vaccine efficacy, and viral susceptibility to monoclonal antibodies including PrEP. Although highly transmissible and resistant to existing monoclonal antibodies, the most recent SARS-CoV-2 variant, Omicron, causes mild-to-moderate COVID-19 in most people, including LTRs (1). However, LTRs with even mild-to-moderate COVID-19 remain at increased risk for progression to severe disease and its resultant morbidity and mortality (1). This increased risk of disease progression is illustrated by our cohort of 113 LTRs with mild-to-moderate COVID-19, as 32 (28.3%) required hospitalization, 13 (11.5%) required ICU admission, 6 (5.3%) required mechanical ventilation, and 12 (10.6%) died of COVID-19, despite early and aggressive treatment.

Remdesivir is an intravenous drug with potent *in vitro* activity against a range of RNA viruses, including MERS-CoV, SARS-CoV-1, and SARS-CoV-2, that interferes with viral replication by prematurely terminating viral RNA transcription (8–10). The Infectious Disease Society of America (IDSA) recommends REM for patients with mild-to-moderate COVID-19 at risk for progression to severe disease (11); these recommendations are based on a clinical trial that showed a reduction in hospitalizations (HR: 0.28; 95% CI: 0.1, 0.75) in patients treated with 3 days of REM initiated within 7 days of symptom onset (12). According to the IDSA, the overall certainty of evidence for treatment of patients with mild-to-moderate COVID-19 was low as less than half of the original projected sample size was enrolled into the clinical trial. However, giving REM early in the disease course appeared to have a robust effect, thus the benefit of treatment likely outweighs the harm in patients at high risk for severe disease. Molnupiravir is an oral pro-drug that is converted to β-D-N4-hydroxycytidine, which acts as a substrate for RNA-dependent RNA polymerase. After β-D-N4-hydroxycytidine is incorporated into the viral RNA, serial mutations develop, resulting in a virus that is less fit for ongoing viral replication. The IDSA recommends MOL for patients with mild-to-moderate COVID-19 at high risk for progression to severe disease, but only if there are no other available treatment options. Notably in June 2023, Merck Sharp & Dohme B.V. withdrew its application for a marketing authorization of MOL from the European Medicines Agency due to an inability to show a clinically relevant treatment benefit on COVID-19 in adults. However, given the logistical challenges of REM administration at our institution, we still transitioned to MOL as soon as it became available, but did so with trepidation given the presumed superiority of REM antiviral therapy.

## Conclusions

As COVID-19 continues to pose a serious threat to LTRs, effective and safe therapeutic agents are imperative. Monoclonal antibody therapy and PrEP have become obsolete due to SARS-CoV-2 viral spike protein mutations rendering these agents ineffective. Furthermore, highly effective antivirals like nirmatrelvir-ritonavir may pose a risk due to serious drug-drug interactions, thereby reiterating the need for safe and potent treatment strategies for LTRs with COVID-19. Our retrospective cohort study of LTRs with mild-to-moderate COVID-19 suggests that oral MOL, a drug with a favorable safety profile, has comparable efficacy to intravenous REM, when adjusted for the SARS-CoV-2 strain, higher rates of vaccination, monoclonal antibody therapy, and PrEP among patients treated with MOL. Although significantly limited by our small sample size, as evidenced by wide confidence intervals, and retrospective nature, we found no differences in the odds of hospitalization, ICU admission, mechanical ventilation, or COVID-19-related mortality between LTRs treated with REM and those treated with MOL on adjusted analyses (Table 3). We also found no difference in clinical outcomes between LTRs who did not receive antiviral therapy and those treated with MOL, which has two plausible explanations. It is possible that MOL does not reduce the risk of hospitalization, ICU admission, mechanical ventilation, or death among LTRs with mild-to-moderate COVID-19, especially among those with the Omicron strain. However, our center treats all patients with mild-to-moderate COVID-19 with antivirals who present within the first 7 days of symptom onset; thus, the group of LTRs that was not treated with antivirals is enriched with patients whose symptoms were so mild that they did not test for COVID-19 or call the transplant center in a timely manner. It is therefore also possible that treatment with MOL equalizes the risk of adverse outcomes between patients with mild-to-moderate COVID-19 and those with very mild disease. Our findings suggest that MOL may be a suitable alternative for REM, irrespective of the SARS-CoV-2 strain, vaccination, or PrEP status. Hence, the choice of antiviral therapy can be driven by practical considerations such as route of administration and drug availability. Larger, multicenter, randomized controlled trials are needed to confirm these findings.

## Data Availability

The raw and de-identified data supporting the conclusions of this article will be made available by the authors, without undue reservation.
